# Development and evaluation of a family-child reading picture book on reducing autism spectrum disorder caregivers’ psychological stress: a mixed method study

**DOI:** 10.3389/fpsyt.2024.1390430

**Published:** 2024-05-08

**Authors:** Lei Yang, Jinlin Ye, Hongrui Zhu, Yao Tang, Xianhong Li

**Affiliations:** ^1^ Xiangya School of Nursing, Central South University, Changsha, Hunan, China; ^2^ School of International Nursing, Hainan Medical University, Haikou, Hainan, China

**Keywords:** autism spectrum disorder, caregivers, psychological empowerment, picture book, ADDIE

## Abstract

**Introduction:**

The rapid increasing prevalence of ASD has become a significant global health issue. Caregivers of children with ASD are experiencing higher level of psychological stress and mental disorders. However, interventions to improve the psychological health of caregivers of children with ASD have largely been neglected.

**Methods:**

Based on the ADDIE (Analysis, Design, Development, Implementation, and Evaluation) model, we initially did in-depth interviews with 8 caregivers, and conducted field observation in two rehabilitation centers to analyze the daily lives, the empowered components, the emotional moments of the children with autism and their caregivers. Then we designed the outline of the picture book, and developed it by a multi-disciplinary team by 4 rounds. After that, this picture book was sent out to 54 caregivers of children with ASD for family-child reading in one month. A quantitative questionnaire was administered before and after their reading to evaluate the efficacy of reducing their stress and affiliate stigma, and improving self-efficacy, resilience, empowerment capacity; and exit interviews were conducted after their initial reading to assess the acceptability, content appropriateness, perceived benefits and generalizability of this picture book. Quantitative data were analyzed by descriptive analysis and paired *t*-tests using IBM SPSS 26.0. Qualitative data were analyzed using template analysis.

**Results:**

In total, 54 caregivers read the picture book with their child, with the total of 149 (an average of 2.76 per family) times reading in one month. Among them, 39 caregivers returned the following-up questionnaires. Although most of the outcome measures did not showed significant changes except the stress level decreased statistically significant (13.38 ± 3.864 to 11.79 ± 3.238, P=0.001), caregivers reported that the picture book echoed their daily lives and gave them a sense of warmth, inspiration, and hope, as well as some insight on family relationships and attitudes towards the disorder. They also expressed a willingness to disseminate the book to other families with children suffering ASD and the public.

**Conclusion:**

This specially designed picture book has been proven to be an acceptable, content-appropriate, and effective family-centered psychological intervention, which could be easily scaled up.

## 1 Introduction

Autism spectrum disorder (ASD) is a heterogeneous neurodevelopmental condition. It is characterized by early-onset difficulties in social communication, and unusually restricted repetitive behavior and interests (1). During the past two decades, the Autism and Developmental Disabilities Monitoring (ADDM) Network estimated that the prevalence of ASD among children at 8 years has increased markedly, from 6.7 (one in 150) per 1,000 in 2000 to 27.6 (one in 36) per 1,000 in 2020 (2). The rapid increasing prevalence of ASD has become a significant global health issue, not only leading to a heavy social and economical burden for the society, but also leading to huge psychological stress for a large number of families with children with ASD (3).

Caregivers of children with ASD were not only more likely to exhibit physical symptoms such as fatigue (4), pain (5), sleep disturbances (6), and other chronic illnesses (7), but also more likely to experience higher level of psychological stress. There was ample evidence to suggest that parents of children with ASD experience significantly higher levels of stress compared to those of typically developing children or children with other neurodevelopmental disabilities (8, 9). Extensive studies have examined that the severity of the child’s illness (10), chronic disruptions in parental routines and heavy family financial burdens (11) perpetuated high levels of parenting stress. Elevated stress levels can have a detrimental impact on caregivers’ mental health, which in turn can adversely affect the development of their children. Therefore, it is important to alleviate the psychological stress of caregivers of children with ASD (12). In addition, studies have shown that caregivers’ resilience (13), family empowerment (14), self-efficacy (12), and affiliate stigma (15) were all correlated with levels of psychological stress.

However, interventions to improve the psychological health of caregivers have largely been neglected (16). Until recent years, some researchers started to design interventions to target caregivers’ psychological well-being (17). The intervention strategies included providing basic information (18), relevant resources and services (19), as well as psychotherapy related intervention, for example, cognitive behavioral therapy (20), acceptance and commitment therapy (21), and positive psychology counseling (22). All of the these intervention strategies offered promising outcomes for improving the psychological health of caregivers. However, the interventions usually were time-consuming and professional-based, which limited the generalization of the intervention strategy. Besides, previous psychological interventions were mostly individual-centered, while family-centered psychological intervention was limited. Considering caregivers’ stress of children with ASD might be influenced by the interactions between family members, as well as the families’ attitude towards the disorder, thus family-centered empowerment interventions might have positive impact on relieving caregivers’ psychological stress.

Picture books, as a type of literary carrier, have brought a new era of psychological empowerment intervention through its beautiful text expression and vivid image portrayal (23). Narrative transportation theory (24) posits that when people are immersed in a story, they experience a high degree of cognitive involvement and emotional engagement, and generate vivid mental representations. This might reshape the readers’ attitude and belief about the reality, thus reduce the psychological stress. According to the double ABCX model (25), parental psychological stress might came from three factors: factor A was stressors, such as the affiliate stigma of caregivers; factor B was the internal and external resources of the family, such as external resources (family empowerment) and internal resources (self-efficacy and psychological resilience); and factor C is coping strategies. Parent-child reading, an activity of a parent reading to a child when both members having their attention focusing on the same text and content (26), has shown benefits for both caregivers and children. The mechanism might lie in that readers could link themselves with the characters in the picture book, experience and learn the coping strategies in the story, which evokes strong emotional echoes for them (27). Therefore, it could potentially reduce psychological stress by empowering the families, reducing affiliate stigma, improving self-efficacy and psychological resilience. Previous studies indicated that parent-child reading could promote children’s oral language, increase children’s reading attention span, and improve reading comprehension (26, 28). Weisleder (29) conducted an innovative program focused on promoting parent-child reading, and results showed that parent-child reading could promote parent-child interactions, and improve children’s language and cognitive development. Nonetheless, Canfield and colleagues (30) concluded that parent-child reading could make caregivers feel “warm” and relieve parenting stress through the promoted parent-child interaction. An study conducted during the COVID-19 period in Brazil stated that parent-child reading buffered parents’ stress resulted from the COVID-19-related events (e.g., income loss, child care disruption) (31).

Therefore, this study aimed to develop a picture book targeting on describing the daily life of a ASD child and how their family members taking care of him, and then examine the efficacy on relieving caregivers’ psychological stress through family-child reading, and evaluate the acceptability, content appropriateness, perceived benefits and generalizability of this picture book.

## 2 Methods

The ADDIE model including “Analysis, Design, Development, Implementation, and Evaluation” phases was adopted to guide this study’s process (32). It has been widely used to effectively develop educational products including traditional and e-learning resources (33). Based on the ADDIE model, the following steps were taken.

### 2.1 Analysis

By conducting field observation and caregiver interviews, we analyzed the daily lives of the children with ASD, the empowered components (e.g., effective coping techniques, positive attitudes) and emotional moments (e.g., feeling achievement, feeling being loved) for family members, and integrated those findings into the stories of the picture book.

#### 2.1.1 Field observation

Three of the researchers conducted non-participant observation in December 2021 at two rehabilitation centers (“Aimeng Rehabilitation Center for Children with Special Needs” and “Ruijian Accessibility Development Center”) for 4 half days. The total observation time was approximately 12 hours. In total around 52 children with ASD and caregivers were present at each observation site during the observation period. Each family usually stayed for two hours in the rehabilitation center for two sessions’ training. We observed not only the children’s emotion, behavior, interaction and communication with others, but also the caregivers’ emotional expressions when they were waiting or observing their kids’ rehabilitation process, or having interviews with the researchers. Typical cases were approached to enrich the understanding. Observation notes with reflections of the researcher were taken to provide practical elements for story creation, in order to ensure the echo of the caregivers when they were reading the story.

#### 2.1.2 In-depth interviews with caregivers

Between December 2021 and January 2022, the purposive sampling strategy was adopted to recruit caregivers of children with ASD for face-to-face in-depth interviews from the two rehabilitation centers. A semi-structured interview guide based on the literature and expert consultation was developed by the research team and piloted in two interviews (included in the final analysis). Questions were open-ended and probes were proposed when needed, covering the following main questions: 1) Please tell me something about your child (e.g., when got the diagnosis, the current situation)? 2) Have you encountered some difficulties during the rehabilitation process? Please describe it. 3) How did you cope with those difficulties? 4) Have you had some cheerful or moved moments? What happened? 5) How do you think about the disorder? 6) What kinds of resources do you need? According to the data saturation principle (34), each theme had at least one typical scenario for developing the picture book story when we interviewed the 8^th^ caregiver, and thus recruitment stopped. Thematic analysis was used to analyze the data, and the results were used to assist the creation of the skeleton and content of the picture book.

### 2.2 Design

Based on the results of the first step, a one-day committee meeting was held to discuss the appropriate title, skeleton of the story, and the main components of knowledge education, rehabilitation skills, interaction techniques, supporting resources, and psychological empowerment for caregivers. The committee members included stakeholders from Disabled Persons’ Federation, rehabilitation centers, hospitals, nursing school of a university, liberal arts college of a university, and a picture book visualization company. During the committee meeting, themes from the interviews, typical cases and real stories were provided to facilitate the design of this picture book.

### 2.3 Development

Initially, the research team developed the script of the picture book. Then, a multi-disciplinary team (MDT) reviewed, discussed and edited the script for 4 rounds, with 2 face-to-face discussion meetings. The MDT included one pediatric nursing expert (PI of this study) and one senior graduate student who had previously participated in developing the other two picture books (35, 36) targeting on children with chronic illnesses, one writer^1^ who had published lots of picture books, and one linguist who was responsible for checking the English translations, one editor who was in charge of the final quality of the script, and three graduate students with two majoring in pediatric nursing and one majoring in art. In addition, during the later stage of the development, we invited three 5-year-old children to read the content and provide relevant suggestions on which expressions were challenging to understand. Finally, a tested version of the picture book was developed.

### 2.4 Implementation

This study adopted a pre-post design. The recruitment flyer was posted at the lobby of the two rehabilitation centers from April 2022 to July 2022. Potential caregivers could contact the researchers if they were interested. The inclusion criteria were: 1) family member of a child diagnosed with ASD aged 3 to 10 years old; 2) taking care of the child for at least one year; 3) able to read and verbally communicate with the child, and 4) volunteering to participate in this study. Those who were involved in other psychological intervention studies were excluded.

The sample size was determined using G*power program (37) with a effect size of 0.5 based on a previous study of reading therapy for children with ASD and their families (38). Considering a 10% attrition rate, a minimum sample size of 38 could have 80% power at 0.05 significance level. Finally, fifty-four caregivers were invited to participate in the study. The caregivers attended a half hour training workshop on family-child reading skills provided by the rehabilitation center’s faculty. The workshop included live demonstrations on how to read the picture book with children, followed by group coaching and discussion (8-10 persons in each group). The picture book was provided to them at the end of the workshop. Then their children were invited to come to read the book together. After that, family-child reading was conducted at home at least 1 time per week, about 10-20 minutes each time, for 4 weeks. They could finish the reading at a pace, time, and space convenient for them, and they could also review as many times as they want. Checking messages were sent every week and feedback were collected to make sure they read the book with their children. All caregivers were contacted by a research assistant on a biweekly basis to promote treatment integrity. Monitoring of treatment integrity relied on caregivers’ self-reports. However, the research team asked them to submit a short reading log or report after each reading, outlining their main thoughts and feelings to check completion.

### 2.5 Evaluation

A convergent parallel mixed methods was conducted. A pre-post design was adopted to evaluate the impact on caregivers’ psychological stress, self-efficacy, resilience, empowerment capacity and affiliate stigma. A semi-structured interview was conducted to collect their perceptions about the acceptability, content appropriateness, perceived benefits and generalizability of this picture book.

Pre-test quantitative data were collected from all caregivers prior to the workshop (T0). The post-test quantitative data were collected at the 4^th^ week (T1). Demographic information included child information (age, gender, type of school attended, place of residence, presence of siblings, cost of treatment, and payment type) and caregiver information (gender, age, relationship to the child, residence, education, marital status, occupation, and monthly income). The outcome indicators were evaluated using the widely used relevant scales:

#### 2.5.1 Primary outcome

##### 2.5.1.1 Psychological Stress: Questionnaire on Psychological Stress of Parents with Disabled Children

Zhang Rongsheng et al. developed Questionnaire on Psychological Stress of Parents with Disabled Children for Chinese families based on the self-assessment questionnaire (Questionnaire on Resource and Stress, QRS). The questionnaire consists of 33 items with 5 dimensions. Each question has two options, “yes” and “no”, with “yes” being scored as “0” and “no” as “1”. The higher score indicates a higher stress level (39, 40).

#### 2.5.2 Secondary outcome

##### 2.5.2.1 Self-Efficacy: The General Self-Efficacy Scale (GSES)

The GSES was developed by Schwarzerand and translated into Chinese by Wang Cai Kang in 2001. It is a 10-item psychological scale with an internal reliability measured by Cronbach’s α coefficient of 0.85. Each item is answered on a 4-point scale, with total score ranging from 10 to 40 (41, 42).

##### 2.5.2.2 Resilience: The Connor-Davidson Resilience scale (CD-RISC)

The CD-RISC was developed by Connor and Davidson. Yu Xiaonan translated it into the Chinese version, which included three dimensions of resilience (13 items), self-improvement (8 items) and optimism (4 items). The scale is rated on a 5-point Likert scale, ranging from “never” to “always”. The Cronbach’s α coefficient of the scale is 0.910 (43, 44).

##### 2.5.2.3 Family empowerment: Chinese version of the family empowerment scale (C-FES)

The Family Empowerment Scale (FES) was developed in 1992 by Koren et al. and translated by Long Ying et al. in 2020. It includes 6 dimensions. The total Cronbach’s α coefficient of the scale is 0.937. The scale is rated on a 5-point Likert scale from “very non-conforming” to “very conforming”, with a total score of 32 to 160 (45, 46).

##### 2.5.2.4 Affiliate stigma: Affiliate Stigma Scale (ASS)

The Affiliate Stigma Scale was developed by Mak et al. in 2008. The scale consists of 22 items on a Likert 4-point scale ranging from strongly disagree (1 point) to strongly agree (4 points). Exploratory factor analysis reveals that this scale is a one-dimensional scale with an internal consistency coefficient of 0.95 (47, 48).

Quantitative data were collected through an online professional questionnaire (www.sojump.com) and then directly exported to the statistical software SPSS26.0. The mean and standard deviation were used to describe the continuous variables, and the rate and composition ratio were used to describe the categorical data. Paired samples *t*-test was used to compare differences at baseline and one month post-intervention.

Qualitative data for the 54 caregivers were collected through face-to-face interviews which were conducted immediately after the first family-child shared reading. All the interviews were recorded using audio recordings. Each interview lasted approximately 10-30 minutes. A interview outline was used and covering the following topics: 1) the acceptability of this book; 2) feelings and experiences after reading; 3) perceived benefits after reading; and 4) perceptions and suggestions for generalizability. The questions were flexible, allowing the interviewer to follow caregivers’ answers and prompt for more in-depth information as appropriate. Qualitative data were analyzed using template analysis. It is a method often applied to the analysis of qualitative data to systematically analyze textual data by identifying predefined codes or themes and organizing them into a structured framework (49, 50). The template analysis model proposed by Brooks (51) was applied through the following four steps (52): 1) “A priori” themes were established based on our interview aims [**Supplementary Table 1**]. 2) Extract the content of the interviews and summarize them by category. Not all recordings could be transcribed considering the limited time and the analytical nature of the template analysis. When transcripts were not available, analysts summarized interviews directly from the recordings by listening to the interviews and extracted information into the template. 3) The team held meetings every week. Sub-themes were developed, dropped or modified based on discussions. 4) Finally, a set of themes and sub-themes were agreed upon by all researchers. The interview data were put into the template matrix according to the main themes and sub-themes for an exhaustive and specific analysis.

### 2.6 Ethical code

The study was approved by the Institutional Review Board of the Xiangya School of Medicine, Central South University (#202111013). The caregivers were informed about the purpose and significance of the study, the rights and obligations, risks and benefits, etc. All the caregivers signed informed consent forms and volunteered to participate in this study.

## 3 Results

### 3.1 Analysis on the field observation and in-depth interview

The age of the children in the rehabilitation center was mainly between 3-6 years old. There were around 28 children having rehabilitation training in “ Aimeng” Center, and around 26 children in “Ruijian” center every day. Observations yielded evidence about the typical characteristics of children with ASD, caregivers’ interactions with the children, and emotional struggles. We conducted in-depth interviews with 8 caregivers, including 3 males and 5 females. Among them, 4 interviewees were grandparents and 4 were mothers. The average age of the caregivers was 48 years (range of 31-67) [**Table 1**]. Thematic analysis generated five categories including: typical characteristics of children with ASD, delayed diagnosis, emotional struggling, new techniques and getting rewarded. The detailed findings were illustrated in **Supplementary Table 2**.

**Table 1:** Characteristics of the caregivers in the analysis phase(N =8).

Number	Age	Gender	Education level	Occupation	Relationship with children
01	67	Male	Junior college	Retired	Grandfather
02	58	Female	Primary school	Retired	Grandmother
03	57	Male	High school	Retired	Grandfather
04	35	Female	College	Architect	Mother
05	38	Female	College	Educator	Mother
06	61	Male	Middle school	Retired	Grandfather
07	31	Female	Junior college	Stay-at-home	Mother
08	34	Female	Junior college	Stay-at-home	Mother

### 3.2 Design and development of the picture book

Based on the above analysis, the multidisciplinary team identified the specific needs of psycho-education and the empowered components for the families suffering from ASD. The components obtained from the observations and interviews guided us in designing and developing the picture book. For example, caregivers sometimes exhibited emotional manifestations such as fidgeting, sighing, scowling, or even tearing when interacting with the child during the observation period. In the interview, caregiver similarly expressed emotional problems (08 “I couldn’t eat or drink, I couldn’t sleep at night. I was very anxious and my families were all in chaos”). Therefore, in the design and development phase, caregiver characters who use problem-centred solutions were portrayed in the story. It can inspire readers to abandon the emotion-centred reactions, cherish the grateful or cheerful moments, and thus reduce stress. The details about how the findings from the analysis been used to guide the creation of the picture book were illustrated in **Supplementary Table 2**. The first column was the main themes; the second column was the sub-themes, and followed by the evidences from observation (column 3) and interviews (column 4); the fifth column was the purpose of integrating such elements into the story, and the two column were the exemplars of the description and illustration of the story. Then, the multidisciplinary team developed the story script both in Chinese and English, and the painter drew the pictures vividly. The draft picture book titled “My Brother” consisted of 48 pages with color illustrations, and in comic book format of 787*1092 mm. The story depicted the growth of a child with ASD from the perspective of the elder sister, who shared the heartwarming story of how the family helped the brother “adjust to life on earth”. Because the girl’s character could psychologically empower the caregivers much better than a “parent character” due to her naive love. The story was followed by educational information for the public and some social supporting resources for the families.

### 3.3 The evaluation of the picture book

A total of 54 caregivers from two rehabilitation centres volunteered to participate in our study between May and September 2022, and all of them finished the baseline survey and the immediate face-to-face interview after their initial reading, and 39 (72.2%) caregivers finished the one-month follow-up survey. The caregivers consisted of 36 women and 3 men with an average age of 36.62 ± 7.58 years old (range=29 to 64). Most of them (33/39) were mothers, and more than half were full-time caregivers (25/39, 64.1%). The majority had a monthly income between 5,000 - 9,999 RMB (725–1,449 USD, 14/39, 35.9%), but the treatment and rehabilitation cost also ranged between 5,000 - 9,999 RMB (725–1,449 USD) per month. Their children had an average age of 5.49 ± 1.73, 33.3% (13/39), and most did not attend school for education and only 18% (7/39) attended inclusive kindergarten or primary school. More detailed characteristics were described in **Table 2**.

**Table 2:** Characteristics of the participants in the evaluation phase(N =39).

Variable	n (%)
Gender	
Male	3 (7.7)
Female	36 (92.3)
Age	
26-30	3 (7.7)
31-40	30 (76.9)
41-50	3 (7.7)
51-60	2 (5.4)
Over 60	1 (2.6)
Relationship with children	
Farther	2 (5.1)
Mother	33 (84.6)
Maternal grandmother	1 (2.6)
Paternal grandfather	1 (2.6)
Paternal grandmother	1 (2.6)
Other	1 (2.6)
School the child attends	
Inclusion kindergarten	4 (10.3)
Normal kindergarten	19 (48.7)
Inclusion primary school	3 (7.7)
Not attending school	13 (33.3)
Having siblings	
Yes	23 (59.0)
No	16 (41.0)
Location	
Countryside	9 (23.1)
City	30 (76.9)
Education level	
Primary school	1 (2.6)
Middle and high school	16 (41.0)
Junior college	14 (35.9)
College	7 (17.9)
Post-Graduate	1 (2.6)
Marital status	
Married	37 (94.9)
Divorced	2 (5.1)
Occupation	
Corporate employees	2 (5.1)
Business employees	2 (5.1)
Individual business	2 (5.1)
Freelance	4 (10.3)
Stay-at-home	25 (64.1)
Other	4 (10.3)
Monthly income (RMB/USD)	
≤999/≤145	5 (12.8)
1,000-2,999/146–434	5 (12.8)
3,000-4,999/435–724	10 (25.6)
5,000-9,999/725–1,448	14 (35.9)
≥10,000/≥1,449	5 (12.8)
Costs for rehabilitation treatment (RMB/USD)	
≤999/≤145	1 (2.6)
1,000-2,999/146–434	9 (23.1)
3,000-4,999/435–724	6 (15.4)
5,000-9,999/725–1,448	12 (30.8)
≥10,000/≥1,449	11 (28.2)
Payment method for rehabilitation treatment	
Self-Pay	17 (43.6)
State or institutional partial subsidy	20 (51.3)
other	2 (5.1)

#### 3.3.1 Efficacy

The quantitative pre-post evaluation showed that caregivers’ psychological stress statistically decreased (T0 = 13.38 ± 3.864, T1 = 11.26 ± 3.210, P=0.000). The caregivers’ self-efficacy (T0 = 22.15 ± 3.682, T1 = 22.28 ± 4.478, P=0.839), resilience (T0 = 57.49 ± 14.136, T1 = 57.54 ± 13.826, P=0.969), family empowerment (T0 = 120.13 ± 12.622, T1 = 121.92 ± 12.278, P=0.233) and affiliate stigma (T0 = 40.49 ± 7.608, T1 = 39.97 ± 7.506, P=0.513) did not change significantly [**Table 3**].

**Table 3:** Means, standard deviations, and paired t test analysis of outcome variables.

Measure	Baseline	Post-test	t	p
	Time 0	Time 1		
	Mean ± SD	Mean ± SD		
Psychological Stress(Questionnaire on Psychological Stress of Parents with Disabled Children)	13.38 ± 3.864	11.26 ± 3.210	4.377	<.001^*^
Self-Efficacy(GSES)	22.15 ± 3.682	22.28 ± 4.478	-0.205	.839
Resilience (CD-RISC)	57.49 ± 14.136	57.54 ± 13.826	-0.040	.969
Family Empowerment (C-FES)	120.13 ± 12.622	121.92 ± 12.278	-1.213	.233
Affiliate Stigma(ASS)	40.49 ± 7.608	39.97 ± 7.506	0.660	.513

^*^ p< 0.001; p values < 0.05, were considered statistically significant.

#### 3.3.2 Acceptability & appropriateness

According to the self-report of caregivers, a total of 10 people completed all the planned readings (first reading and 4 readings of T0 to T1) and 27 people completed 3 or more times. The total number of readings was counted as 149 with an average of 2.76 times per person (about 3 times). Most of them (32/54) believed this picture book vividly described a “warm” and “inspiring” story. They echoed their similar or exact experiences, the tough events and the cheerful moments in the book. They said they like to “read” it, and their children like to “watch” it. Overall, caregivers’ feedback suggested that picture book was quite acceptable and appropriate. **Table 4** illustrated the summarized domains and themes.

**Table 4:** Themes on interviews with caregivers after reading the picture book.

Domain	Themes
Appropriateness & Acceptability	Feeling “warm” and be “loved”
	Feeling inspiring and hope
	Feeling the echo of their life
	Appropriate supporting resources
Perceived Benefits	Learned the value of family members’ support
	Learned the significance of siblings and how to balance the love
	Learned to accept
	Learned to be confident
Generalizability	Recommend this picture book to special families
	Recommend this picture book to the public

##### 3.3.2.1 Feeling “warm” and be “loved”

Caregivers said they could perceive “warm” and “love” in the story of the picture book. As Caregiver 04 said, “After reading, I feel that this is a story book making me gentle in my inner world.” Even some caregivers expressed their emotions by covering their faces or sobbing while reading the “mess” situations in the family (Caregiver 38), some were moved to tears when feeling the whole family’s love for the child (Caregiver 17). As Caregiver 50 said, “Obviously, although the younger brother was diagnosed with ASD, his family members gave him all the love they could, rather than abandoning him. And it was rewarded since the child made big progress. Love is powerful. This story should be seen for every family with a child with ASD.”

##### 3.3.2.2 Feeling inspiring and hope

Some were feeling “cheerful” for the progress that the child made in the story, which brought some kinds of hope in their own lives (Caregiver 05,17,48). As Caregiver 29 stated, “In the first month after the intervention, he improved rapidly. For the first time, he knew what ‘love’ meant. He showed what love was by kissing and hugging me. After reading, I felt sure that my child would become better and better, which inspired me to work harder to earn money for supporting his rehabilitation.” Besides, the warm and helpful relationship between a sibling and the child with ASD depicted in the story also encouraged the family to move on. Caregiver 08 said, “The relationship between the elder sister and the younger brother was very heartwarming to me. This relationship inspired me that maybe my child really needed a sibling’s company (she originally planned not to have a second child).”

##### 3.3.2.3 Feeling the echo of their life

Most caregivers perceived the story as vivid descriptions of their daily lives. The child’s performance, daily life, recovery process and psychological state portrayed in the story resonated with the real life of them. Caregiver 49 stated, “It completely depicts our mindset, our thoughts. It’s very similar to our family, like telling our own story.” Caregiver 32 said, “My child’s symptoms are exactly the same (as the character), such as emotional problems and restricted repetitive behaviors. This story is very realistic.”

##### 3.3.2.4 Appropriate supporting resources

Some caregivers expressed excitement about learning about the supportive resources in the appendix. They indicated that the related support resources in the picture book provided families with access to learning and getting social support. As Caregiver 46 stated, “I can read the information in this appendix. I think it is quite useful.” One caregiver praised the picture book for its “scientific description”, and said, “the first step to accept the disorder is to know the scientific nature of the disorder, and this book provided it” (Caregiver 50).

#### 3.3.3 Perceived benefits

The template analysis for the qualitative data yielded positive feedback [**Table 4**]. Some caregivers emphasized their perceived benefits from reading this picture book.

##### 3.3.3.1 Learned the value of family members’ support

Most caregivers realized that supportive family relationships played an important role in the growth of their children. Most caregivers reported that the picture book prompted them to rethink their family relationships and changed their attitude towards this disorder and the child. They stated that they would be more willing to make some changes for a harmonious family relationship, such as “participating in rehabilitation throughout” (Caregiver 14), “expressing love more” (Caregiver 17), and “accompanying more” (Caregiver 21). As Caregiver 23 stated, “In order to build a warm family atmosphere for my children, the whole family moved to Changsha. We spend time with our child in rehabilitation, which led to his rapid progress. The benefits of a warm family are endless.”

Most female caregivers indicated that the picture book made them realize the importance of emotional support from fathers. Since in the story, all the empowerment ideas were from the father. In the traditional culture belief, fathers usually took the financial responsibility and were rarely involved in their children’s rehabilitation and emotional companionship, which they perceived as “unfair” (Caregiver 26). Caregivers expressed encouragement for fathers to be more involved in the family to avoid the children missing out on fatherhood. As Caregiver 28 stated, “It is important to encourage men to be more involved in family and share the psychological stress of the counterpart. Although my kid is not perfect, yet who knows that he might make big progress when his father participated more in the family.”

##### 3.3.3.2 Learned the significance of siblings and how to balance the love

Caregivers learned the significance of siblings and how to balance the love for the siblings. The picture book was written from the perspective of a sister. The sister brought the love and companionship different from the parents and was indispensable in her brother’s growth. They indicated that the siblings assisted the child with daily tasks, took care of him or her in the absence of parents, and guided the child to follow some “social rules” (Caregiver 30). They described the children’s mutual help and attachment, but also mentioned competition and neglect. Caregivers indicated that they tent to neglect their other children (without ASD) due to the increased needs of children with ASD. Extra effort should be made to balance the love for both children. As Caregiver 50 stated, “I feel I’m stuck in a dilemma. On the one hand, I want my kids to live as free as they want. On the other, I would ignore the needs of my general child, and want him to be able to take care of his brother (with ASD) when I pass away. After reading, I realize that it is very important to balance the love of two children.”

##### 3.3.3.3 Learned to accept

Most of the families learned “acceptance of this special child”. They demonstrated this urgent need not only for the society, but also the families. As Caregiver 17 stated, “What I learned from this picture book are acceptance. If we can’t accept our children, how can we hope the society will remove the misconceptions about them.”

##### 3.3.3.4 Learned to be confident

Caregivers indicated that this story brought them confidence in consistent rehabilitation. Caregivers said that the child’s rehabilitation was a life-long, persistent and all-encompassing challenge. It was important to intervene early and persist. As Caregiver 07 said, “This picture book has encouraged me not to give up, and to intervene early with my child so that he can get better (outcomes).”

#### 3.3.4 Generalizability

Many caregivers (23/54) indicated that they would like to distribute this picture book to other families with children with ASD, since “they are suffering unimaginable stress” (Caregiver 35). As Caregiver 54 described, “Actually, it is possible (for me to recommend this book to others). Many families are suffering from tremendous psychological pressure, and this book can be of help.” Besides, some caregivers also recommended the public, especially the normally developing children and their families could read it. They wished the public could pay more attention to the family suffering from ASD. As Caregiver 50 stated, “children with ASD were more likely to experience ‘adverse childhood experiences’ than other more visible types of disability or general children. Promote the picture book to the public so that the public would know about ASD, recognize it, and eventually accept it, thus reducing their rejection and discrimination towards children with ASD and their families.”

## 4 Discussion

Guided by ADDIE model, this study rigorously developed and evaluated a picture book aiming to empower families with children with ASD through family-child reading. The development of the picture book was multidisciplinary and evidence-based to guarantee the quality of the content and illustrations. The mixed methods evaluation results confirmed its acceptability, content appropriateness, generalizability and efficacy on reducing psychological stress.

The ADDIE model is widely applied to systematic and instructional program design in teaching and it is a novel, interdisciplinary approach for designing educational programs (53). The ADDIE model helps researchers create learner-centered interventions ensuring the feasibility and acceptability (54). This study illustrated the rigorous process of developing a psychologically empowered picture book according to the ADDIE model. In this study, we identified the common needs and priorities of caregivers through field observation and in-depth interviews, which were commonly used in designing other educational programs (55, 56). As in Chen’s study (57), the blended emergent research training program developed based on needs assessment ultimately showed a positive impact on nurses’ research competencies and critical thinking. Eui Geum Oh and his colleagues (58) also confirmed that the needs assessment-based education program for heart failure patients was well understood and operable. Studies have shown that due to its adaptability, ADDIE could facilitate and meet most instructional needs (59). This may have been a key factor in receiving such positive comments from caregivers in our study, because the findings from the analysis phase were fully used for developing the picture book. For example, to alleviate caregivers’ psychological stress and emotional struggles, the overall tone of the story was designed to be warm and full of love; to address caregivers’ “helplessness”, we added illness-related knowledge and social support resources following the story. A multi-disciplinary team with rich experience is also essential for designing and developing well-informed, systematic, and effective content. This study emphasized the collaboration of multiple researchers from different disciplines, which helped to remove inherent disciplinary barriers, enabled the integration of a wide range of knowledge (60), and ensured the reliability of the book content. Ultimately, the positive results in the summarized evaluation further support the usefulness and integrity of the ADDIE model.

This study’s psychological intervention takes the “family-centered” perspective by choosing family-child reading approach. There are three rationals. The first consideration is that children with ASD cannot “read” the picture book by themselves, but many of them are interested in “watching” and “reading”, and thus a reading guider is needed for these children. The second consideration is that family members could be the optimal reading guider for their children, since they could find flexible learning time and space. The third consideration is that family-child reading could also promote their interaction and emotional echoes with their children. Therefore, this picture book through family-child reading, as a simple, effective, and family-centered intervention, has been proven to be an acceptable, flexible, and comfortable for families with children with ASD by our study. Also similar to previous studies, family-centered interventions were associated with many positive parent, family, and child outcomes. It overall can lead to greater family satisfaction, stronger self-efficacy beliefs within the family, increased family involvement, greater family empowerment, and improved child behavior and functioning (61).

The results of the study showed that the shared reading of the picture book by caregivers and children was effective in relieving the psychological stress of the caregivers. Previous studies have shown similar results. In Garcia’s (62) study, picture books were distributed to preoperative children and their parents and the result showed it reduced preoperative anxiety of children and relieved psychological stress of parents. Caregivers in our study expressed two major benefits from reading the picture book: establishing supportive family relationships and reshaping the cognition towards the reality. First of all, we found that the parental roles and family relationships in the story had served as role models for most families. As previous studies have shown, high levels of family relationship quality could promote positive parenting (63), prevent psychological problems (64) and reduce psychiatric disorders (65). In addition, caregivers learned about the important role of fathers in the rehabilitation process of children with ASD. A growing number of researchers have begun to emphasize the important role of fathers’ involvement in children development (66). Fathering input can positively influence young children’s intellectual development (67), social emotion (68), and behavior problems (69). Besides, when raising a child with ASD, there are a variety of challenges for caregivers. However, our team acknowledged such difficulties, but emphasizing the psychological gains and growth of caregivers (70), which could be regarded as post-traumatic growth (PTG) (71). Post-traumatic growth is not a direct result of trauma, but rather the coping strategies used in dealing with trauma (71). Zhang (72) adopted a solution-focused brief therapy with mothers of children with ASD and reported increased PTG scores. Likewise, our study found that caregivers gradually changed their perceptions of the child and focused more on the positive aspects, such as keeping faith in the future and accepting the child’s differences. The picture book improves the PTG of caregivers. We cannot deny that time may have played a role in the caregivers’ cognitive change, but some caregivers indicated that the change was happened after reading the picture book. Therefore, the picture book can be used in the future as a cost-effective measure to help readers re-establish rational cognitive patterns and improve the way they cope with negative events, thus promoting psychological growth.

Although the qualitative evaluation received positive feedback, not all the quantitative indicators showed statistically significant changes before and after the intervention. There might be some possible reasons. First of all, the intensity of the intervention may not be sufficient. The recommended reading frequency for caregivers and children is once a week, and only for 4 weeks; besides, there is only one story and the content intensity may not be enough. Second, the mental health indicators we measured, such as resilience, are relatively stable. It is difficult for them to change within a short period of only one month. However, the qualitative feedback data confirmed the potential capacity to improve their resilience by reshaping their cognition towards this disorder. Third, the children’ rehabilitation progress is the main concern of the family, which largely affect the caregivers’ mental health. However, because of the wide spectrum of autism, the severity and rehabilitation progress of each child are very different. Not all of the families noticed rewarded progress from their children in one month’ period.

There are several limitations of this study that should be considered. First, the story we depict reflects the real lives and experiences of children with ASD and their families as much as possible. However, due to the wide spectrum of autism, it is difficult for us to match everyone’s expectations. The story describes a boy who makes huge progress through rehabilitation with the love of his family. Some caregivers viewed this story ending as “idealistic” for their child. Therefore, we need to take full account of the different practical situations faced by most families of children with ASD in future studies, and consider to develop serious of such kinds of picture books. Second, this study only evaluated the efficacy of the picture book by using a pre-post design, more rigorous study designs could be considered in future studies. Moreover, the feedback of children with ASD hasn’t been included into the evaluation for the sake of communication barriers. But their reactions could be valuable data too. In future studies, we can consider observing their performance after reading and communicating with their caregivers. From cultural perspective, the themes and content of this picture book are based on the experiences and feelings of the general family of children with ASD rather than culture-specific experiences. In addition, the picture book is written in bilingual Chinese and English with clear and simple illustrations. Thus, the picture book would be able to be resonated and understood by readers from different cultural backgrounds. In future research, we need to involve and incorporate more culturally competent elements to enhance readers’ empathy and truly achieve psychological empowerment.

## 5 Conclusion

This specially designed picture book has been proven to be an acceptable, content-appropriate, and effective family-centered psychological intervention, which could be easily scaled up among families with children with autism. It could also be recommended to the public as educational material and reduce people’ prejudice towards ASD. In future, scholars and clinical healthcare providers could develop picture books that target the psychological empowerment of children with different types of disabilities and their families. Moreover, this type of psycho-educational approach could also been validated among various types of childhood illnesses, not only for enhancing knowledge and coping techniques, but also for improving children and their families’ mental health well-being.

## Data availability statement

The raw data supporting the conclusions of this article will be made available by the authors, without undue reservation.

## Ethics statement

The studies involving humans were approved by The Institutional Review Board of the Xiangya School of Medicine, Central South University. The studies were conducted in accordance with the local legislation and institutional requirements. Written informed consent for participation in this study was provided by the participants’ legal guardians/next of kin.

## Author contributions

LY: Writing – review & editing, Writing – original draft, Visualization, Formal analysis, Data curation. JY: Writing – review & editing, Investigation, Data curation. HZ: Writing – review & editing. YT: Writing – review & editing. XL: Writing – review & editing, Supervision, Resources, Methodology, Funding acquisition, Conceptualization.

## Footnotes

1 Sulan Tang , female, a first-class writer in China, a professor at the Liberal Arts College of Hunan Normal University and the president of the Hunan Provincial Writers Association. She has written and published more than 40 works of children’s literature, and has won a number of prestigious literary awards in China.
