# Supplementary material for: Development and evaluation of a family-child reading picture book on reducing autism spectrum disorder caregivers’ psychological stress: a mixed method study
Source: Front Psychiatry. 2024 May 8;15:1390430. doi: 10.3389/fpsyt.2024.1390430 (PMC11165404; doi:10.3389/fpsyt.2024.1390430)
Supplement: Supplementary file 1 [file DataSheet_1.docx]

Supplementary Material

# Supplementary Table

**Supplementary Table 1. Data extraction template（Priori）**

| **Domain** | **Themes** |
| --- | --- |
| Acceptability | Feeling “warm” and be “loved” |
|  | Feeling close to their life |
| Content appropriateness | Same clinical presentation |
|  | Progress in rehabilitation |
|  | Love and support in the family |
| Effectiveness | Family relationship |
|  | Mental health |
|  | Rehabilitation skills and supporting Resources |
| Generalizability | Recommend to special families |
|  | Recommend to the public |

**Supplementary Table 2. Results of the analysis and exemplars of the design and development of the picture book**

| **Categories** | **Themes** | **Observation** | **Interview quote** | **Purposes of integrating the elements into the story** | **Story description (exemplar)** | **Illustrations (exemplar)** |
| --- | --- | --- | --- | --- | --- | --- |
| Typical characteristics of children with ASD | Social interaction disorders | - Most of the children always refused to engage in playing activities with other children. - When teachers greeted the children with physical gestures, they avoided the teacher’s gaze and did not make eye contact. - When teachers attracted the children with cute toys and motivated them intentionally during teaching activities, they were silent and ignore them. | He never says hello to anyone and never meets anyone’s eyes. (02) | - To resonate with the reader and attract them to the story scenario by depicting the typical characteristics of children with ASD - social interaction disorder. | My brother was born, but he didn’t seem to like me. He wouldn’t even look at me when I called his nickname warmly; he cried loudly when I tried to hug him. | **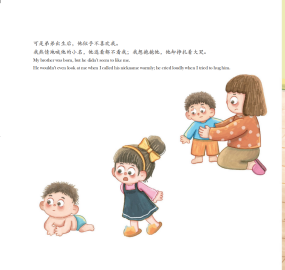** |
|  | Social communication disorders | - When greeted by teachers and other caregivers, the children did not respond with words and might only show their body movements. - During teaching activities, the children never answered questions asked by the teacher. They might look down and pick their fingers, mumble to themselves, or even cry and scream. | Once something upsets her, she likes to scratch her hair instead of expressing it verbally. (07) | - To resonate with the reader and attract them to the story scenario by depicting the typical characteristics of children with ASD - social communication disorders. | But when he was two years old, he still wouldn’t look at me in the eyes when I talked to him. It seemed he didn’t like Mum and Dad either, and he never called “Dad” or “Mum”. | 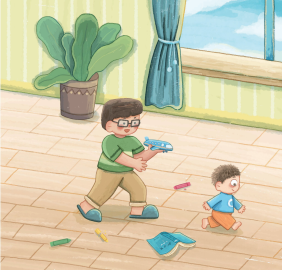 |
|  | Repetitive stereotyped behaviors | - The children liked to repeat what the teacher said, resulting in failure to communicate. - The children preferred to sit in the same seat, eat the same kind of food, and walk along the same route. | She had to sit on the purple chair at home every time and mumbling a commercial sentence showing on TV over and over again. This makes me think she has good memory and I’m still very pleased. (07) | - To resonate with the reader and attract them to the story scenario by depicting the typical characteristics of children with ASD - repetitive stereotyped behaviors. | He liked playing with his eyelashes, or fiddling with his toy train all day, mumbling and babbling from time to time. | **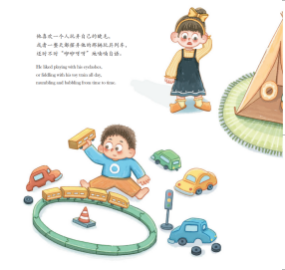** |
|  | Developmental disorders | - The children required constant parental support during the rehabilitation process, otherwise they would be restless. - The children required adult assistance in dressing, undressing, toileting, eating and other activities. | He is completely unable to take care of himself. He can not do activities such as dressing or going to the toilet. (01) | - To resonate with the reader and attract them to the story scenario by depicting the typical characteristics of children with ASD - developmental disorders. | Mum and Dad taught him to recognize things in our daily life, get dressed, go to the toilet, pronounce words and speak... | **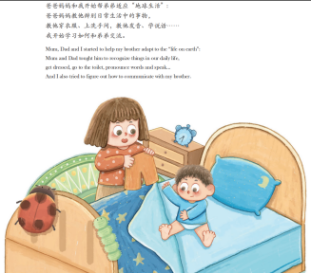** |
| Delayed diagnosis | Lack of knowledge | - Some of the caregivers of children with ASD who came to the rehabilitation centre for counselling showed limited understanding, confusion, and helplessness about their child’s illness. | The child was over 4 years old and still not talking. I didn’t think there was wrong with my child. I didn’t even know what ASD was. (03) | - To publicize the knowledge about ASD by describing the definition, epidemiological characteristics, and clinical manifestations of ASD. - To help caregivers better understand and cope with their children’s situations. - To help caregivers to establish correct cognition, avoid misunderstandings and prejudices, and face their children’s differences with a more inclusive and understanding attitude. | Autism appears in infancy and early childhood and is a representative disorder of the pervasive developmental disorders. | **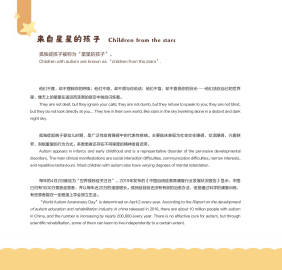** |
|  | Influenced by traditional belief |  | According to the traditional belief, children who talk late are more intelligent. So I didn’t think he was a child with a disease . (02) | - To teach how to identify the early symptoms of ASD to assist caregivers in gaining a better understanding of their child’s condition at an early stage. - To provide psychological support for caregivers. Through early identification, caregivers can seek help and support more effectively, alleviating inner anxiety and stress. | No/Less Seeing, No/Less Responding, No/Less Referring, No/Less Language, Inappropriateness | **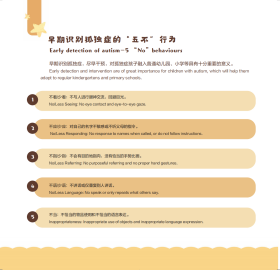** |
| Emotional struggling | Sorrow and denial | - The caregivers sometimes exhibited emotional manifestations such as fidgeting, sighing, scowling, or even tearing when dealing with the child. | For two months after my child was diagnosed I couldn’t eat or drink, I couldn’t sleep at night. I was very anxious and my families were all in chaos. (08) | - To empower the caregivers by establishing a character who adopts positive and problem-focused coping style instead of emotion-focused coping style. - To assist caregivers in effectively managing their challenges, enhancing parenting skills, and maintaining their own mental well-being. | Dad said to me, “Your brother is from the star. He loves Dad, Mum, and you. He just doesn’t know how to express it. Do you want to help him adapt to the ‘life on earth’? | 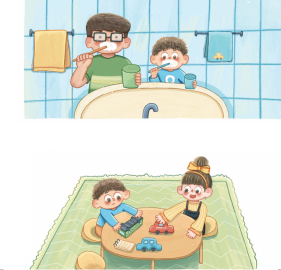 |
|  | Self-blame |    - Some caregivers of children with ASD who came to the rehabilitation centre for counselling said that their children’s suffering from ASD must be related to the fact that they had less time to spend with them and had limited communication with them. | Before I knew I was pregnant, I took the pill. When my child was diagnosed with ASD, I especially blamed myself. I thought it was all my fault. (05) | - To publicize the knowledge about ASD by describing the definition, epidemiological characteristics, and clinical manifestations of ASD. - To help caregivers better understand and cope with their children’s situations. - To help caregivers to establish correct cognition, avoid self-blame. | Autism appears in infancy and early childhood and is a representative disorder of the pervasive developmental disorders. The main clinical manifestations are social interaction difficulties, communication difficulties, narrow interests, and repetitive behaviour. Most children with autism also have varying degrees of mental retardation. | **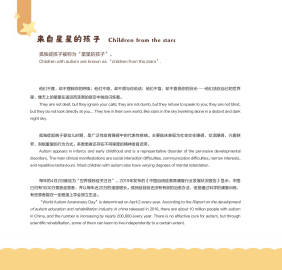** |
|  | Helpless | - We could hear the yelling and the criticism from the caregivers in the hallway and we could get a sense of irritable and helpless of the caregivers. | I don’t know where to apply for financial support from some social organizations or government organizations. (04) | - To provide practical and feasible methods and resource support to promote the healthy growth and development of children with ASD. - To assist in counseling, financial support, rehabilitation services, and psychological support to help them feel less isolated and helpless when facing the challenges. | Children with autism are easily agitated by changes and stimulus, and they may shake their heads, scream, or even attack others. Try to empathize with them, give them enough sense of security, and patiently teach them how to vent their emotions. | **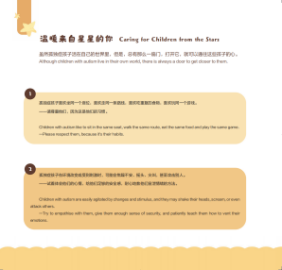** |
|  | lack of family emotional support |  | The power that family brings me is endless. I am also very helpless when my children are disobedient. If I get encouragement from my family at such times, I will be very happy. (04) | - To empower the caregivers by depicting a “warm and loving” family to emphasize the importance of the family environment in a child’s upbringing. - To help caregivers adjust their mindset to face the process of their children’s growth with a more optimistic and resilient attitude. - To provide caregivers with a learning model. They can learn practical skills and methods by observing and imitating the interaction between family members. | My brother is happy, because Dad, Mum, and I all love him. I am also happy, because Dad, Mum, and my brother all love me. My brother and I are growing up in different worlds, but love connects us. | **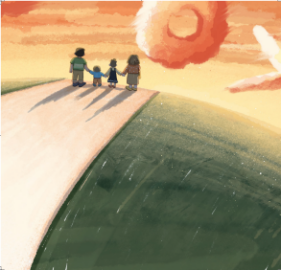** |
| New techniques | Spending more time for outdoor activities |  | I found that as soon as he entered the natural environment, it was as if a switch was flipped. Every time we went to the countryside to play, his ability of expression suddenly improved. (01) | - To empower the caregivers by providing the technique about taking the child to have outdoor activities which would largely improve the child’s progress. | We ran in the country fields and the pleasant wind was blowing into our faces. My brother’s footsteps seemed much lighter than usual. Suddenly, he turned around, looked me in the eyes and said, “I am so happy!” | **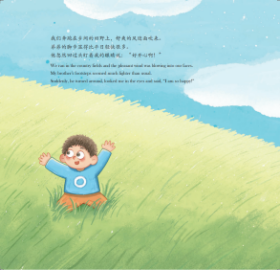** |
|  | Emphasizing father’s involvement | - At the rehabilitation center, about half of the caregivers were their mothers, and half were their grandparents, and only three children were accompanied by their fathers during the observation. | I gave up my job to be with my child full time. My husband left the city to find a job that earned more money. So, the child lacks the love of the father all the time. (08) | - To empower the caregivers by depicting a “responsible” father and emphasize the importance of the father’s role in the child’s recovery. - To provide a behavioral model for fathers in real life. | Dad said to me, “Your brother is from the star. He loves Dad, Mum, and you. He just doesn’t know how to express it. Do you want to help him adapt to the ‘life on earth’?” I nodded solemnly. I loved my brother. Of course，I wanted to help him. | **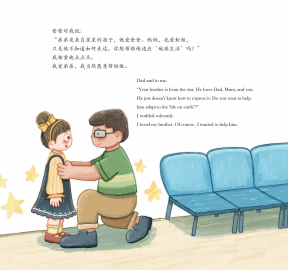** |
|  | Balancing love for siblings |  | We have another elder child at home who is a normal child. I hope he can take care of the child with ASD. However, we pay very less attention to him, and I know I’m sorry for him. (04) | - To help caregivers better navigate the challenges of the parenting process, ensuring that every child receives fair and appropriate care, and promoting the healthy development of family harmony and parent-child relationships. | Although Dad and Mum spent a lot of time with my brother, they never ignored me. And sometimes they specially set aside their own time to accompany me. I felt that I was valued and cherished, too. | **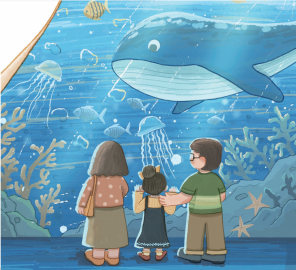** |
| Getting rewarded | Child’s progress |  | Before she was 6, she didn’t care what you were telling at all. Now you talk to her about rules, she can understand some. My child’s progress makes me feel joyful, as if everything I’ve worked for has been rewarded. (06) | - To empower the caregivers by depicting an “idealized” end, in which the ASD child made big progress, and bringing some hope and confidence to move forward. | My brother gradually made great progresses and finally got into a regular kindergarten. One day, Dad and I went to pick up my brother from kindergarten. He was so happy to see us, and he jumped for joy, “Coming! Coming!” On the way home, I tried to hold his hand, but he didn’t resist this time. How warm and soft his little hand was! | **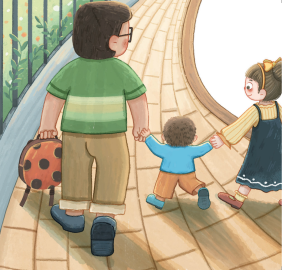** |
